# Supplementary material for: Mechanisms governing the pioneering and redistribution capabilities of the non-classical pioneer PU.1
Source: Nat Commun. 2020 Jan 21;11:402. doi: 10.1038/s41467-019-13960-2 (PMC6972792; doi:10.1038/s41467-019-13960-2)
Supplement: Supplementary file 7 — Source data [file 41467_2019_13960_MOESM7_ESM.zip › Source_Data/FigureS2/FigureS2B_MotifScanOutput/KmeansCluster11/homerResults/motif2.similar.html]

motif2

## Information for motif2

C
T
A
G
A
T
C
G
A
C
G
T
A
C
G
T
A
G
T
C
G
A
T
C
A
T
C
G
A
G
C
T
C
A
G
T
A
C
G
T
A
G
C
T
  
Reverse Opposite:  

T
C
G
A
T
G
C
A
G
T
C
A
T
C
G
A
T
A
G
C
C
T
A
G
C
T
A
G
C
G
T
A
C
G
T
A
T
A
G
C
G
A
T
C
  

|  |  |
| --- | --- |
| p-value: | 1e-174 |
| log p-value: | -4.009e+02 |
| Information Content per bp: | 1.639 |
| Number of Target Sequences with motif | 712.0 |
| Percentage of Target Sequences with motif | 21.91% |
| Number of Background Sequences with motif | 2760.7 |
| Percentage of Background Sequences with motif | 6.11% |
| Average Position of motif in Targets | 101.5 +/- 46.5bp |
| Average Position of motif in Background | 98.3 +/- 67.3bp |
| Strand Bias (log2 ratio + to - strand density) | -0.1 |
| Multiplicity (# of sites on avg that occur together) | 1.05 |
| Motif File: | file (matrix) reverse opposite |

### Similar de novo motifs found

|  |  |  |  |  |  |  |  |
| --- | --- | --- | --- | --- | --- | --- | --- |
| Rank | Match Score | Redundant Motif | P-value | log P-value | % of Targets | % of Background | Motif file |
| 1 | 0.819 | C G T A T C G A C G T A T C G A T A G C C T A G T C A G T G C A C T G A T A G C G C A T A T C G | 1e-98 | -226.533349 | 12.03% | 3.14% | motif file (matrix) |
| 2 | 0.791 | T G C A C G T A T C G A T G A C T C A G T A C G G T C A G C T A | 1e-75 | -174.586682 | 37.18% | 22.25% | motif file (matrix) |
